# Exosome-mediated microRNA signaling from breast cancer cells is altered by the anti-angiogenesis agent docosahexaenoic acid (DHA)

**DOI:** 10.1186/s12943-015-0400-7

**Published:** 2015-07-16

**Authors:** Bethany N. Hannafon, Karla J. Carpenter, William L. Berry, Ralf Janknecht, William C. Dooley, Wei-Qun Ding

**Affiliations:** Department of Pathology, University of Oklahoma Health Sciences Center, Oklahoma City, OK 73104 USA; Department of Cell Biology, University of Oklahoma Health Sciences Center, Oklahoma City, OK 73104 USA; Department of Surgery, University of Oklahoma Health Sciences Center, Oklahoma City, OK 73104 USA; Peggy and Charles Stephenson Cancer Center, Oklahoma City, OK 73104 USA

## Abstract

**Background:**

Docosahexaenoic acid (DHA) is a natural compound with anticancer and anti-angiogenesis activity that is currently under investigation as both a preventative agent and an adjuvant to breast cancer therapy. However, the precise mechanisms of DHA’s anticancer activities are unclear. It is understood that the intercommunication between cancer cells and their microenvironment is essential to tumor angiogenesis. Exosomes are extracellular vesicles that are important mediators of intercellular communication and play a role in promoting angiogenesis. However, very little is known about the contribution of breast cancer exosomes to tumor angiogenesis or whether exosomes can mediate DHA’s anticancer action.

**Results:**

Exosomes were collected from MCF7 and MDA-MB-231 breast cancer cells after treatment with DHA. We observed an increase in exosome secretion and exosome microRNA contents from the DHA-treated cells. The expression of 83 microRNAs in the MCF7 exosomes was altered by DHA (>2-fold). The most abundant exosome microRNAs (let-7a, miR-23b, miR-27a/b, miR-21, let-7, and miR-320b) are known to have anti-cancer and/or anti-angiogenic activity. These microRNAs were also increased by DHA treatment in the exosomes from other breast cancer lines (MDA-MB-231, ZR751 and BT20), but not in exosomes from normal breast cells (MCF10A). When DHA-treated MCF7 cells were co-cultured with or their exosomes were directly applied to endothelial cell cultures, we observed an increase in the expression of these microRNAs in the endothelial cells. Furthermore, overexpression of miR-23b and miR-320b in endothelial cells decreased the expression of their pro-angiogenic target genes (PLAU, AMOTL1, NRP1 and ETS2) and significantly inhibited tube formation by endothelial cells, suggesting that the microRNAs transferred by exosomes mediate DHA’s anti-angiogenic action. These effects could be reversed by knockdown of the Rab GTPase, Rab27A, which controls exosome release.

**Conclusions:**

We conclude that DHA alters breast cancer exosome secretion and microRNA contents, which leads to the inhibition of angiogenesis. Our data demonstrate that breast cancer exosome signaling can be targeted to inhibit tumor angiogenesis and provide new insight into DHA’s anticancer action, further supporting its use in cancer therapy.

**Electronic supplementary material:**

The online version of this article (doi:10.1186/s12943-015-0400-7) contains supplementary material, which is available to authorized users.

## Background

Docosahexaenoic acid (DHA, 22:6) is a long-chain omega-3 polyunsaturated fatty acid and the main component of dietary fish oil that has many health benefits, including anticancer activity [1, 2]. The anticancer properties of DHA have been demonstrated both *in vitro* [3, 4] and *in vivo* [5–7]. Importantly, DHA is cytotoxic to tumor cells, with little or no effects on normal cells [3, 8]. Currently, several clinical trials are evaluating DHA supplementation for breast cancer therapy and management (clinicaltrials.gov). These studies underline the potential value of DHA as both a safe preventative agent and as an adjuvant to therapy.

One of the reported anticancer mechanisms of DHA is the ability to suppress tumor angiogenesis. For example, a DHA-supplemented diet suppresses tumor angiogenesis as measured by microvessel counts in a breast cancer nude mouse model [9] and this observation was confirmed in a murine mammary tumor model also fed a fish oil diet [10]. The anti-angiogenic activity of DHA is also described in a human colon cancer model system [11], a fibrosarcoma implantation model in Fischer 344 rats [12], and in human umbilical cord vein endothelial cells [13]. The cellular mechanisms of how DHA suppresses tumor angiogenesis remain unclear. Traditionally, vascular endothelial growth factor (VEGF), which is secreted from cancer cells in response to hypoxia, is considered the key regulator of tumor angiogenesis and current strategies to inhibit tumor angiogenesis are primarily focused on targeting the VEGF pathway [14]. However, recent studies have demonstrated that other cellular signaling molecules, such as exosomes, also mediate tumor angiogenesis [15–17].

Exosomes are small (50–100 nm) vesicles that have recently been recognized as important mediators of intercellular communication. They carry lipids, proteins, mRNAs and microRNAs that can be transferred to a recipient cell [18, 19]. Tumor cells have been shown to secrete exosomes in greater amounts than normal cells [20], thus allowing the transfer of tumor-associated signaling molecules to surrounding cells [21–23]. Importantly, the microRNAs in secreted exosomes can be transferred to a recipient cell where they affect post-transcriptional gene regulation [24]. Cancer cell-derived microRNAs can be transferred via exosomes to endothelial cells where they induce pro-angiogenic effects [15, 16]. These studies underline the role tumor-derived exosomes can play in the tumor microenvironment and in promoting tumor angiogenesis. However, very little is known about the contents and secretion of breast cancer exosomes or ways to manipulate or reduce their influence on cancer progression. In this study we sought to determine how DHA might alter the secretion and contents of breast cancer exosomes thereby suppressing tumor angiogenesis and progression, which may lead to a better understanding of breast cancer biology and novel strategies targeting intercellular communication for breast cancer therapy.

## Results

### DHA increases the small RNA contents of breast cancer exosomes

**Fig. 1:**
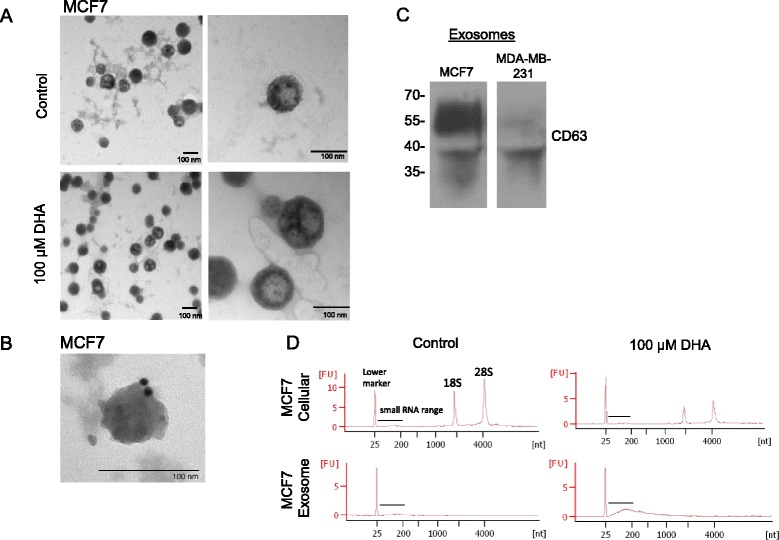
Isolation and molecular characterization of exosomes shed from breast cancer cells. **a** Exosomes were isolated from the conditioned media of control or treated MCF7 cells (100 μM DHA for 24 h) using the Exoquick Reagent, fixed, whole-mounted on coated Formvar grids, negatively stained and visualized by electron microscopy (Hitachi H-7600). Left column, 40,000×; right column 100,000×. Images are representative of three experiments. **b** Immunogold labeling and electron microscopy of human CD63 (10 nM gold particles) on exosomes isolated from MCF7 cells (100,000×). **c** Western blot analysis of the exosome marker CD63 (30–60 kDa) in exosomes shed from MCF7 and MDA-MB-231 cells. Exosome lysates were run under non-reducing conditions. **d** RNA Agilent Bioanalyzer profile of total RNA extracted from control and DHA treated MCF7 cells and their purified exosomes. The peak at 25 nt is a lower marker standard and is indicated on the upper left image. In the exosome preparations the RNA is predominantly <1,000 nucleotides in length, with a large peak noted in the small RNA range of the DHA-treated exosomes and the 28S and 18S ribosomal subunit RNAs (as indicated on upper left image) are notably absent from both treated and untreated exosome preparations

Human breast cancer MCF7 and MDA-MB-231 cells were grown in cell culture medium supplemented with exosome-depleted serum for 3 days and treated with 100 μM DHA for 24 h. Whole exosomes were collected from the conditioned medium of DHA-treated and untreated MCF7 cells. The exosomes were fixed and negatively stained for visualization by electron microscopy. We found exosomes of the typical ~100 nm diameter in both samples (Fig. 1a), (MDA-MB-231 exosomes not shown). The isolated exosomes had detectable CD63 expression, a well-established marker for exosomes, by immunogold labeling and imaging by electron microscopy (Fig. 1b) and by western blot (Fig. 1c). Total RNA was isolated from the exosomes and a RNA profile was generated. We found that the RNA isolated from the MCF7 exosomes was primarily small RNA of less than 1,000 nt and typically absent of ribosomal RNA as compared to the total cellular RNA (Fig. 1d). These observations are similar to previous reports [25, 26]. However, in the DHA treated exosome RNA sample we observed an increase in the small RNA content of <200 nt (see Fig. 1d, lower right panel). These results indicate that DHA increases the small RNA content of breast cancer exosomes.

### DHA increases microRNA levels in breast cancer cells and exosomes

To further determine if DHA increases the small RNA content of breast cancer secreted exosomes, a small RNA library was prepared from equal quantities of total RNA isolated from the DHA-treated and untreated MCF7 exosomes and cells. The resulting cDNA library was sequenced and the reads were mapped onto the human genome (Build 36, Mar 2006) and intersected with known mature microRNAs (mirBase, v13; www.mirbase.org). Overall, DHA treatment increased the number of mature microRNAs detected in the MCF7 cells and exosomes. In the cells, 387 microRNAs were detected (at least 1 read) in control MCF7 cells versus 412 detected in the DHA-treated MCF7 cells (increase of 25 microRNAs). In the exosomes, 196 microRNAs were detected in the control exosomes versus 209 detected in the DHA-treated exosomes (increase of 13 microRNAs) (Additional file 1). These results are consistent with the increase in small RNA content observed in the RNA profile (Fig. 1d). Twenty-six cellular microRNAs were changed (1.5-fold or greater) by DHA-treatment (19 up-regulated and 7 down-regulated) (see control cell vs. DHA cell in Additional file 1). Three microRNAs (miR-1246, 204-fold; miR-451, 135-fold; and miR-127-3p, 6-fold) were preferentially encapsulated into the exosomes and found at very low levels in the cellular content, which is in agreement with a recent study [27] (see control cell vs. control exosome in Additional file 1). In the DHA-treated exosomes 91 mature microRNAs were detected (at least 1 read), 83 of which were changed by 2-fold or greater compared to the untreated exosomes and 22 of these microRNAs were present at greater than 1,000 copies in at least one condition; these 22 microRNAs are listed in Table 1. Our results demonstrate that DHA alters the microRNA profile of MCF7 cell-derived exosomes.

**Table 1:** DHA increases microRNA expression levels in MCF7 exosomes

microRNA	Mapped reads		Fold-change	P-value
	Control	DHA		
miR-23b	205.5	1064.8	5.18	1.0E-20
miR-378	333.3	1510.2	4.53	1.0E-20
let-7f	2949.3	11386.4	3.86	1.0E-20
miR-21	10853.1	41786.2	3.85	1.0E-20
let-7a	4748.9	17764.0	3.74	1.0E-20
miR-182	2044.0	6986.1	3.42	1.0E-20
miR-151-3p	522.1	1760.1	3.37	1.0E-20
miR-27b	4082.4	13189.9	3.23	1.0E-20
let-7e	472.1	1521.1	3.22	1.0E-20
miR-27a	4032.4	12896.6	3.20	1.0E-20
let-7c	449.9	1434.2	3.19	1.1E-16
miR-365	888.7	2824.9	3.18	1.0E-20
miR-320b	516.6	1553.7	3.01	1.0E-20
let-7i	783.2	2335.9	2.98	1.0E-20
miR-30d	955.3	2814.0	2.95	1.0E-20
miR-26a	1122.0	3302.9	2.94	1.0E-20
miR-30a	994.2	2890.0	2.91	1.0E-20
miR-200b	810.9	2314.2	2.85	1.0E-20
miR-181a	399.9	1086.5	2.72	7.5E-11
miR-191	3204.8	7507.6	2.34	1.0E-20
miR-151-5p	938.7	2151.2	2.29	1.0E-20
miR-1246	4537.9	9072.1	2.00	1.0E-20

The microRNAs with a fold-change ≥ 2 and mapped reads ≥1,000 in at least one condition are shown. The fold change was calculated by normalizing the reads to the number of mapped reads and the p-values were calculated using the Likelihood Ratio Test and adjusted using the Benjamini and Hochberg post-test

### DHA increases let-7a, miR-21, miR-23b, miR-27b, and miR-320b levels in exosomes from other breast cancer cell lines, but not from normal breast cells

**Fig. 2:**
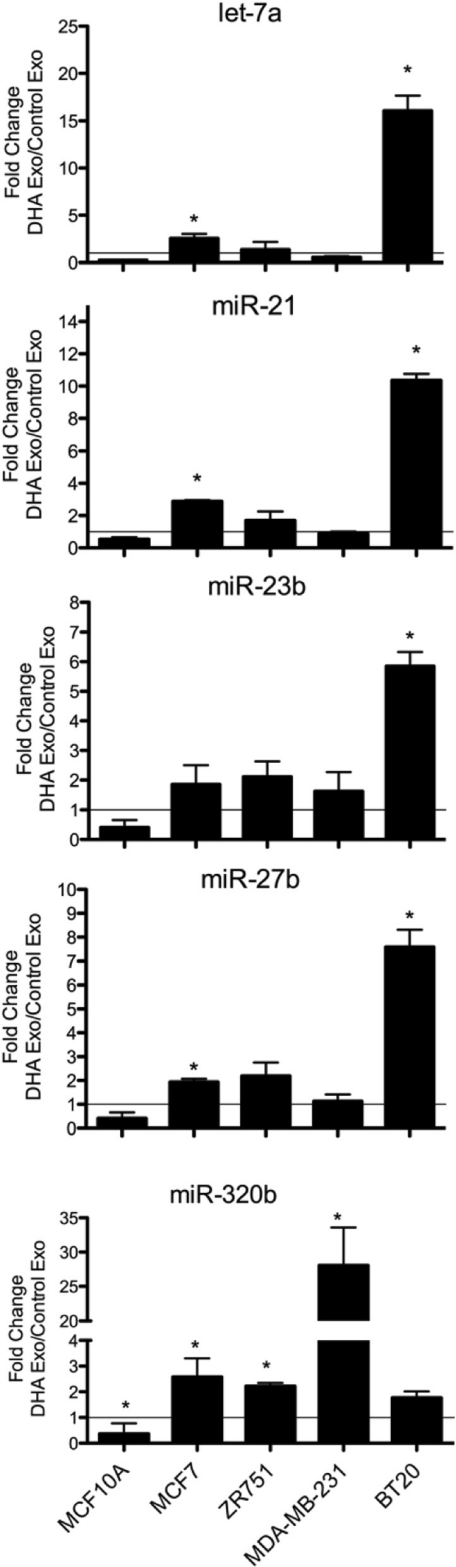
Validation of DHA-induced microRNA expression changes in exosomes secreted from breast cancer cells. MCF10A, MCF7, ZR751, MDA-MB-231 and BT20 breast cancer cells were plated at a density of 5x10^6^ cells per 150 mm dish and cultured for 3 days. Cells were treated with 100 μM DHA for 24 h. Exosomes were collected from the conditioned media by ultracentrifugcation, total RNA was extracted, reverse transcribed and amplified by real-time PCR using microRNA specific primers (*n* = 3, error bars = SE). The fold change in expression levels was calculated using the ΔΔCt method. *p < 0.05 by Student’s t test

To determine if the effects of DHA on exosome microRNA levels are specific to only MCF7 breast cancer cells or whether DHA can increase the exosome microRNA levels in normal mammary epithelial cells or other breast cancer types, we cultured a normal mammary epithelial cell line (MCF10A), an ER-positive luminal line (ZR-75-1) and two triple-negative basal-like breast cancer lines (BT20 and MDA-MB-231). Exosomes were isolated from the conditioned media after DHA treatment and total RNA was extracted from the control and DHA-treated exosomes. The expression of 5 of the most abundant microRNAs (let-7a, miR-21, miR-23b, miR-27b, and miR-320b) detected in the DHA-treated MCF7 exosomes was measured in the exosomes from the other breast cell lines by qRT-PCR. As shown in Fig. 2, DHA treatment increased the expression of all 5 of these microRNAs in the DHA-treated exosomes as compared to control exosomes, with the exception of let-7a and miR-21 in MDA-MB-231 cells. In contrast, DHA-treatment did not increase the expression of these microRNAs in the exosomes from MCF10A cells; rather microRNA expression was decreased by DHA-treatment in these exosomes. These results indicate that the effect of DHA on the microRNA content of their exosomes is specific to cancer cells and is not dependent on the breast cancer subtype.

### DHA increases exosome secretion from breast cancer cells

**Fig. 3:**
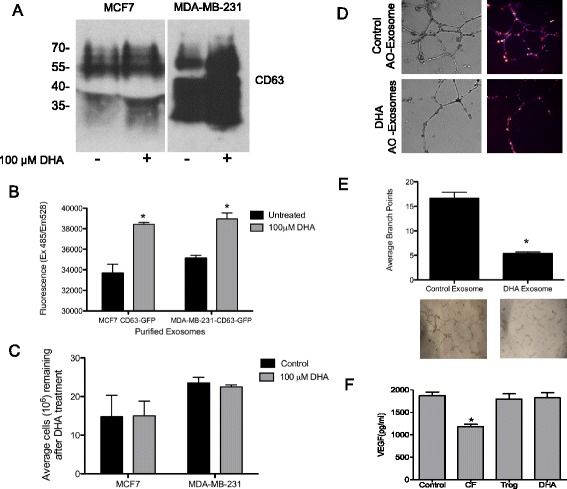
DHA increases exosome secretion from breast cancer cells and reduces tube formation by endothelial cells without affecting VEGF secretion. **a** Western blot analysis of CD63 expression in exosomes isolated from MCF7 and MDA-MB-231 cells after treatment with DHA. Equal volumes of exosome protein lysate were loaded and SDS-PAGE was run under non-reducing conditions. **b** CD63-GFP tagged exosomes secreted into cell culture medium from DHA-treated or untreated MCF7 and MDA-MB-231 breast cancer cells were isolated by ultracentrifugation, re-suspended in PBS and the GFP levels were measured by fluorescent spectrometry, * *p* < 0.0001 using one-way ANOVA (*n* = 3). **c** MCF7 and MDA-MB-231 breast cancer cells were treated with 100 μM DHA for 24 h. The media was removed for exosome isolation and the breast cancer cells that remained after DHA treatment were counted and are expressed as the average cell number per 10^6^ cells (*n* = 3, error bars = SE). **d** MCF7 cells were treated with 100 μM DHA for 24 h, the exosomes from the treated and untreated cultures were isolated from the culture media by ultracentrifugation and stained with acridine orange (AO). The AO-labeled exosomes (100 μg) were incubated with EA.hy926 cells (1×10^4^) seeded on ECMatrix in a 12-well plate for 24 h and imaged with a Perkin Elmer Operetta at 40× magnification, bright field *(left)* and fluorescence *(right),* 460 nm excitation and 650 nm emission. **e** Approximately 1/10 of the exosomes isolated from a confluent 100 mm plate of DHA-treated or untreated MCF7 cells were mixed with 1×10^4^ EA.hy926 cells and seeded on ECMatrix in a 96-well plate and incubated for 18 h. Tube formation was visualized with a light microscope (*lower*) and counted (*upper*) *, *p* < 0.001 using one-way ANOVA (*n* = 4). **f** MCF7 cells were treated with 100 μM DHA, 1 mM clofibrate (CF) or 20 μM troglitazine (Trog) for 24 h and VEGF in the medium was analyzed by ELISA and expressed as pg/ml. *, *p* < 0.001 using one-way ANOVA (*n* = 4-6)

To determine if exosome secretion is altered by DHA we measured CD63 expression in DHA-treated and control exosomes secreted from MCF7 and MDA-MB-231 cell lines by western blot. We found increased levels of CD63 protein in the DHA-treated exosome preparations from both cell lines (Fig. 3a). In order to monitor exosome production and measure their release we generated MCF7 and MDA-MB-231 cell lines stably expressing the exosome marker CD63 tagged with GFP (CD63-GFP) (Additional file 2). The MCF7 and MDA-MB-231 CD63-GFP cells were grown in cell culture media supplemented with exosome-depleted FBS and treated with 100 μM DHA for 24 h. Exosomes were isolated from the conditioned media by ultracentrifugation and suspended in PBS. The presence of GFP in the isolated exosomes was measured by fluorescent spectrometry. We observed a significant increase in exosome secretion from DHA treated versus untreated cell cultures (Fig. 3b), while the cell number was unaffected by DHA treatment (Fig. 3c). These results indicate that DHA increases exosome secretion from breast cancer cell lines.

### Breast cancer exosomes are absorbed by endothelial cells and DHA treatment inhibits endothelial cell tube formation without affecting VEGF levels

We then asked whether the anti-angiogenesis activity of DHA could be due to the DHA-induced changes in exosome secretion and microRNA contents that we had observed. First, to determine if breast cancer exosomes can be readily transferred to endothelial cells we cultured the endothelial cell line EA.hy926 on matrigel in the presence of purified exosomes isolated from MCF7 cells that were pre-labeled with the nucleic acid binding dye, acridine orange (AO). The purified AO-labeled exosomes were applied to EA.hy926 cells and imaged by fluorescent microscopy. The presence of AO-exosomes in endothelial cells was observed as early as 2 h (not shown) and 24 h (Fig. 3d) indicating that breast cancer exosomes are readily transferred to endothelial cells. Then to determine if tube-formation (*in vitro* angiogenesis) by endothelial cells is affected by the DHA-induced changes in the breast cancer exosomes, equal volumes of exosomes isolated from control and DHA-treated MCF7 cells were applied to cultures of EA.hy926 plated on matrigel. Tube formation by EA.hy926 cells was significantly inhibited by the exosomes purified from DHA-treated MCF7 cells compared to those purified from control MCF7 cells (Fig. 3e). These results suggest that DHA suppresses tumor angiogenesis via modulating breast cancer cell-derived exosome contents. This conclusion was further supported by the observation that DHA and troglitazone (a PPARγ ligand) did not alter VEGF secretion, while clofibrate (a PPARα ligand) reduced VEGF secretion from MCF7 cells (Fig. 3f).

### microRNAs released by DHA-treated breast cancer cells are transferred to endothelial cells

**Fig. 4:**
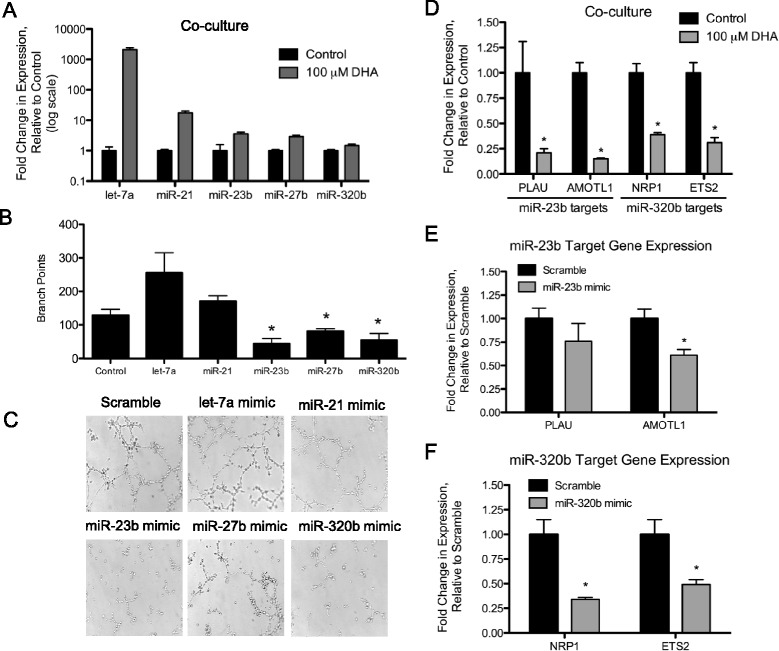
DHA-induced exosome microRNA transfer from MCF7 cells to endothelial cells alters microRNA expression, endothelial tube formation, and target mRNA expression. **a** EA.hy926 cells (2.5×10^4^) were plated in a 24-well plate and MCF7 cells (2×10^4^) were plated in a transwell insert (0.4 μM) and co-cultured for 24 h. MCF7 cells were treated with 100 μM DHA for 24 h, RNA was extracted and qRT-PCR was performed. The fold change in expression levels was calculated using the ΔΔCt method. Data in bar graphs represent mean ± SEM (*n* = 3). EA.hy926 cells were transfected with a scramble control, let-7a, miR-21, miR-23b, miR-27b, or miR-320b mimics. After 48 h EA.hy926 cells (1×10^4^) were plated on ECMatrix in a 96-well plate and incubated at 37 °C for 16–18 h. Branch points were counted (**b**) and representative images (**c**) were taken and under a light microscope (20×). Shown are the combined triplicate results from three independent experiments. Data in bar graphs represent mean ± SEM (*n* = 9). *, *p* < 0.001 using Student’s t test. **d** EA.hy926 cells (2.5×10^4^) were seeded on a 24-well plate and MCF7 cells (2×10^4^) were cultured overnight in a transwell insert (0.4 μm). MCF7 cells in the upper chamber were treated with DHA for 24 h. RNA was extracted from the EA.hy926 cells in the lower chamber and target gene expression was measured by qRT-PCR. EA.hy926 cells were transfected with a scramble control, miR-23b, or miR-320b mimic. The previously validated mRNA targets (PLAU and AMOTL1) for miR-23b (**e**) and (NRP1 and ETS2) miR-320b (**f**) were measured by qRT-PCR 48 h post-transfection. The fold change in expression levels was calculated using the ΔΔCt method. Data in bar graphs represent mean ± SEM (*n* = 3). *, *p* < 0.05 using Student’s t test

Several of the most abundant microRNAs in the DHA-treated exosomes have known activity in targeting endothelial cells and suppressing angiogenesis, such as miR-21 [28], miR-23b [29], miR-27b [30] and miR-320b [31]. Therefore, it is highly likely that these microRNAs contribute to the anti-angiogenesis activity of DHA. To test whether these microRNAs can be absorbed by endothelial cells, MCF7 cells were co-cultured with EA.hy926 cells for 24 h and the MCF7 cells were treated with DHA. RNA was extracted from the endothelial cells and the expression levels of these microRNAs were measured by qRT-PCR. As shown in Fig. 4a the expression of let-7a, miR-21, miR-23b, miR-27b, and miR-320b were dramatically increased in the endothelial cells by DHA treatment of the MCF7 cells. A similar microRNA expression pattern was obseved when DHA-terated exosomes were directly applied to EA.hy926 cells (Additional file 3). These results indicate that the microRNAs carried by the DHA-treated breast cancer cell exosomes are readily transferred to endothelial cells.

### miR-23b, miR-27b, and miR-320b inhibit tube formation by endothelial cells

To determine which microRNA cargo carried by the MCF7 exosomes might modulate the anti-angiogenesis effects of DHA we transfected EA.hy926 cells with microRNA mimics for miR-21, miR-23b, miR-27b, miR-320b, let-7a or scramble control. microRNA overexpression in the EA.hy926 cells was confirmed with at least a 10-fold increase in expression as determined by qRT-PCR 48 h post-transfection. Following microRNA mimic transfection the cells were plated on matrigel and cultured. Tube formation by the endothelial cells was quantitated by branch-point counting (Fig. 4b) and imaged by light microscopy (Fig. 4c), as we have previously described [32]. As shown, increased levels of miR-23b, miR-27b, and miR-320b in the endothelial cells dramatically reduced tube formation by the endothelial cells, whereas overexpression of let-7a and miR-21 did not reduce the tube formation, suggesting that the exosomal microRNAs, miR-23b, miR-27b, and miR-320b, mediate DHA’s anti-angiogenesis activity.

### Co-cultivation of breast cancer cells or microRNA mimic transfection of miR-23b or miR-320b inhibits pro-angiogenesis target mRNA expression

Several previously defined and validated target genes of miR-23b and miR-320b are known to encode proteins that modulate angiogenesis, including plasminogen activator (PLAU) [33], angiomotin like-1 (AMOTL1) [34], neuropilin 1 (NRP1) [31], and v-ets avian erythroblastosis virus E26 oncogene homolog 2 (ETS2) [35]. We asked whether exosome transfer of miR-23b and miR-320b to endothelial cells would affect expression of these pro-angiogenesis target genes. To address this, we co-cultured EA.hy926 cells with MCF7 cells for 24 h. As shown in Fig. 4d, treatment of the co-cultured MCF7 cells with DHA dramatically decreased the expression of PLAU, AMOTL1, NRP1 and ETS2 in the endothelial cells. Direct application of microRNA mimics to the EA.hy926 cells for miR-23b repressed the expression of its target genes PLAU and AMOTL1 (Fig. 4e) and mimics for miR-320b repressed the expression of its target genes NRP1 and ETS2 (Fig. 4f). These results demonstrate that the DHA-induced changes in the microRNA contents of breast cancer secreted exosomes, particularly the increase in miR-23b and miR-320b, can suppress the expression of pro-angiogenesis microRNA target genes.

### Rab27A knockdown reverses DHA-induced exosome-mediated microRNA transfer and inhibition of tube formation by endothelial cells

**Fig. 5:**
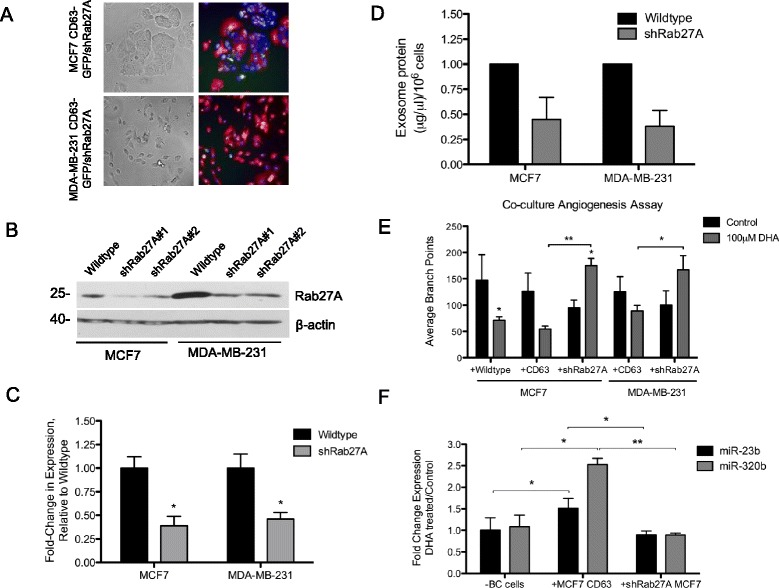
Rab27A knockdown in breast cancer cells inhibits DHA-induced exosome transfer. The expression of Rab27A was knocked down in MCF7 CD63-GFP and MDA-MB-231 CD63-GFP breast cancer cell lines by transduction with a lentivirus expressing dsRed2 and two different shRNAs targeting Rab27A. **a** Bright field (*left*) and fluorescent microscopic images (*right*) of CD63-GFP (green), shRab27A-dsRed2 (red) and Hoescht nuclear stain (blue) (40× magnification). Rab27A protein and mRNA knockdown was confirmed by western blot (**b**) and qRT-PCR (**c**). The fold change in expression levels of Rab27A relative to an endogenous control was calculated using the ΔΔCt method. Data in bar graphs represent mean ± SEM *, *p* < 0.001 using Student’s t test. **d** The quantity of exosomes secreted from breast cancer cells was measured by BCA assay and represented as the μg/μl of whole exosome per 10^6^ cells, relative to wildtype. Data in bar graphs represent mean ± SEM (*n* = 2). **e** MCF7 and MDA-MB-231 cells expressing CD63 or shRab27A-CD63 were grown in a transwell insert (0.4 μm) and co-cultured with EA.hy926 cells plated on ECMatrix for 24 h. Following treatment of the breast cancer cells in the upper chamber with 100 μM DHA for 24 h the number of branch points formed by the EA.hy926 cells were counted. Data in bar graphs represent mean ± SEM (*n* = 3-6) *, *p* < 0.05; **, *p* < 0.01 using Student’s t test. **f** MCF7 cells expressing CD63 or shRab27A-CD63 were grown in a transwell insert (0.4 μm) and co-cultured with EA.hy926 cells plated in a 24-well plate. Following treatment of the breast cancer cells in the upper chamber with 100 μM DHA for 24 h total RNA was extracted from the EA.hy926 cells, reverse transcribed and expression of miR-23b and miR-320b was measured by qRT-PCR. The fold change in expression levels was calculated using the ΔΔCt method. Fold-change values are normalized to control sample and expressed relative to no breast cancer cells (−BC). Data in bar graphs represent mean ± SEM (*n* = 3) *, *p* ≤ 0.05; **, *p* < 0.001 using Student’s t test

To confirm that the observed effects of DHA on the endothelial cells are dependent on breast cancer exosome secretion and contents, we knocked down the expression of the small Rab GTPase, Rab27A. Rab27A has been shown to control multivesicular endosome membrane docking at the plasma membrane and exosome secretion [36]. The expression of Rab27A was knocked down using a miR-30 based shRNA entry vector with dsRed2 co-expression. The shRab27A-dsRed vector was transduced into MCF7-CD63-GFP and MDA-MB-231-CD63-GFP cells. Expression of the shRab27A construct was confirmed by detection of dsRed levels by fluorescent microscopy (Fig. 5a). Knockdown of the Rab27A protein and mRNA was also confirmed by western blot analysis (Fig. 5b) and qRT-PCR (Fig. 5c), respectively. As shown in Fig. 5d exosome secretion was significantly reduced by Rab27A knockdown. To determine if the effects of DHA on endothelial cell tube formation are dependent on Rab27A-mediated exosome secretion, EA.hy926 cells were seeded on matrigel and co-cultured with the MCF7 CD63-GFP, shRab27A-MCF7-CD63, MDA-MB-231 CD63-GFP, or shRab27A-MDA-MB-231-CD63 cells plated in a transwell insert. DHA treatment reduced tube formation by the EA.hy926 cells when co-cultured with the wildtype or CD63-GFP breast cancer cells. However, branch-point formation was increased by DHA-treatment in the shRab27A co-cultures (Fig. 5e), suggesting that the anti-angiogenesis effects of DHA are dependent on Rab27A-mediated exosome secretion. To test whether the transfer of miR-23b or miR-320b occurs with Rab27A knockdown, EA.hy926 cells were plated on the surface of a 24-well plate and cultured alone or co-cultured with MCF7 CD63-GFP or shRab27A MCF7 CD63-GFP cells and treated with DHA for 24 h. As shown in Fig. 5f, the DHA-induced exosome-mediated transfer of miR-23 and miR-320b was inhibited by Rab27A knockdown as compared to CD63-GFP cells. These results suggest that the anti-angiogenesis effects of DHA on endothelial cells are dependent on breast cancer exosome secretion and content transmission.

## Discussion

To our knowledge this is the first demonstration that DHA, a natural anticancer compound, alters breast cancer exosome secretion and microRNA contents, which consequently lead to the suppression of endothelial tube formation. In addition to revealing new mechanisms of DHA’s anticancer action, these novel findings support the concept that breast cancer exosome signaling can be targeted to inhibit tumor angiogenesis.

The importance of exosomes as mediators of cancer progression has been realized in recent years [37]. However, ways to reduce or change their influence on cancer progression has not been demonstrated. In this study we have shown that DHA can alter the microRNA contents and secretion of breast cancer exosomes. We have provided evidence to show that the microRNA contents of cancer-derived exosomes can be altered, and that these changes can influence their biological activity and intercellular communication. These results provide insight into the future possibility of developing new cancer therapeutic strategies targeting exosome secretion and content transmission.

The anti-angiogenic activity of DHA has been known for some time [9, 10, 38], however the precise mechanism is elusive. In this study we have shown that the exosomes isolated from DHA-treated breast cancer cells were enriched with microRNAs that target several genes involved in endothelial cell migration, regulation of capillary formation and angiogenesis. More importantly, application of these exosomes to endothelial cells inhibited endothelial tube formation. These results strongly suggest that DHA’s anti-angiogenesis effects are at least in part mediated through transfer of exosomes. Because exosomes encapsulate and transfer other biologically active proteins, lipids, and mRNAs, it is also likely that DHA may alter other contents of breast cancer exosomes, which may also mediate the anti-angiogenic effects of DHA.

In the present study we showed that co-culture of endothelial cells with DHA-treated breast cancer cells or application of microRNA mimics significantly inhibited tube formation and repressed the expression of pro-angiogenesis target genes, including PLAU and AMOTL1 for miR-23b and NRP1 and ETS2 for miR-320b. In support of these findings, a recent study has demonstrated that exosomal transfer of miR-320 from diabetic cardiomyocytes to endothelial cells resulted in inhibition of angiogenesis and reduction in ETS2 expression [39], thus suggesting that exosomal transfer of miR-320 may regulate angiogenesis in other biological model systems.

The mechanism of how DHA might be affecting exosome secretion and changes in exosome microRNA contents is unknown at this time. It is known, however, that exosomes are enriched in the sphingolipid ceramide, whose formation is regulated by neutral sphingomyelinase (nSMase) [40]. It has been demonstrated that exosome secretion can be stimulated by ceramide and inhibited by treatment with the nSMase inhibitor GW4869 [41]. Over-expression of nSMase can increase extracellular levels of microRNAs, whereas treatment with GW4869 microRNA secretion can be reduced [24]. Another study has shown that DHA treatment of breast cancer cells *in vitro* and through dietary supplementation *in vivo* induces a 30–40 % increase in nSMase activity and ceramide formation; these effects could also be inhibited by addition of GW4869 [42]. Collectively, these studies indicate that DHA may be involved in regulating exosome secretion and microRNA encapsulation through ceramide formation. However, to date no link has been established connecting DHA, exosomes and microRNA secretion. Here we have provided evidence that DHA can induce exosome secretion and alter the microRNA contents of breast cancer exosomes resulting in changes in their biological function. Whether this effect is mediated through nSMase regulation of ceramide production is yet to be determined.

## Conclusions

In conclusion, this study demonstrates that DHA’s anti-angiogenesis action is highly likely to be mediated through stimulation of exosome secretion and alteration of exosome microRNA contents in breast cancer model systems. These results provide new insight into DHA’s anticancer action and further support the use of DHA as an adjuvant for breast cancer therapy.

## Methods

### Cell culture

The human breast cancer cell lines MCF7, MDA-MB-231, BT20 and ZR-75-1 were obtained from the American Type Culture Collection (Manassas, VA). The cells were cultivated in DMEM medium supplemented with exosome-depleted 10 % fetal bovine serum (FBS), 100 IU/mL penicillin and 100 μg/mL streptomycin (Corning/Mediatech, Inc. Manassas, VA). Exosome depleted FBS was prepared by pelleting the exosomes by ultracentrifugation at 100,000 × *g* for 2 h at 4 °C, the resulting supernatant was filtered through a 0.2 μm pore filter and then added to cell culture medium. The EA.hy926 endothelial cell line was kindly provided by Dr. Doris Benbrook (University of Oklahoma Health Sciences Center, Oklahoma City, OK) and was cultivated in F12-K (Corning/Mediatech Inc.) medium supplemented with 10 % fetal bovine serum, 50 mg/mL Heparin (Sigma-Aldrich, St. Louis, MO), 0.05 mg/mL Endothelial Cell Growth Supplement (ECGS; Corning/Mediatech Inc.), and 100 IU/mL penicillin and 100 μg/mL streptomycin (Corning/Mediatech Inc.). Cells were routinely maintained in a humidified chamber at 37 °C and 5 % CO_2_.

### Preparation and application of DHA

Docosahexaenoic acid was analytical grade (Sigma-Aldrich) and a stock solution was prepared by dissolving DHA in molecular grade ethanol at 30 mM and stored at −80 °C under argon gas. For DHA treatment a 5 mM working stock was prepared by dilution with 1.5 mM bovine serum albumin in PBS before adding to cell culture. The final concentration of ethanol in the medium was below 0.5 %. Control cells were treated with vehicle buffer.

### Exosome isolation

Exosomes were isolated utilizing a combination of centrifugation and ultracentrifugation according to [43] and filtration or the Exoquick-TC reagent (System Biosciences, Mountain View, CA) following the manufacturer’s protocol. For ultracentrifugation isolation, conditioned cell culture media was collected and centrifuged at 10,000 × *g* for 30 min at 4 °C, to remove cells and large debris. The supernatant was filtered using a 0.22-μm pore filter and the exosomes were pelleted at 100,000 × *g* for 1 h at 4 °C. The exosome pellet was washed with 10 ml of 1X PBS and pelleted again by centrifugation at 100,000 × *g* for 1 h at 4 °C. The resulting pellet was either suspended in 1X PBS for whole exosome applications or further processed for RNA or protein extraction. Total exosome RNA was extracted using the TRIzol reagent (Invitrogen/Life Technologies, Carlsbad, CA) following the manufacturer’s protocol.

### Western blot analysis

Total exosome protein was prepared by re-suspending the exosomes in RIPA Buffer (50 mM Tris–HCl pH 7.4, 150 mM NaCl, 0.5 % sodium deoxycholate, 1 % NP-40, and 0.1 % sodium dodecyl sulfate) containing 1 mM phenlymethylsulfonyl fluoride, 5 μg/ml leupeptin, 2 μg/ml aprotinin, and 1 μg/ml pepstatin A. About 30–40 μg of protein from each sample was separated on a 10 % SDS-PAGE gel, transferred to a PVDF membrane, and blotted with antibodies against CD63 (Santa Cruz Biotechnology, Santa Cruz, CA), Rab27A (Abnova, Taiwan) or β-actin (Sigma-Aldrich, St. Louis, MO).

### Electron microscopy and immunogold labeling

Exosomes were fixed in 2 % paraformaldehyde. The fixed sample was absorbed onto formvar coated copper grids for 20 min in a dry environment. Samples were then fixed in 1 % glutaraldehyde for 5 min. After being rinsed in distilled water samples were stained with uranyl oxalate for 5 min followed by methyl cellulose uranyl acetate for 10 min on ice. Excess liquid was wicked off of the grid using filter paper, and grids were stored at room temperature until imaging. For immunogold labeling, exosomes were fixed in 2 % paraformaldehyde. Samples were absorbed onto formvar coated copper grids for 20 min in a dry environment. Samples were washed with PBS three times. Samples then underwent four washes in 50 mM glycine followed by a 10 min blocking step. Exosomes were incubated with CD63 primary antibody for 30 min, and then samples were washed in washing buffer six times. Samples were incubated in secondary antibody conjugated to 10 nM gold particles for 20 min. Finally, samples were washed in PBS, stabilized with glutaraldehyde, washed in water, and counter stained with uranyl oxalate and methyl cellulose uranyl acetate. Imaging was done on a Hitachi H7600 microscope.

### CD63 overexpression and Rab27A knockdown

To overexpress GFP-tagged CD63 the lentiviral expression vector pCT-CD63-GFP was obtained from System Biosciences, Mountain View, CA. To reduce the expression of Rab27A, shRNAs were cloned into the miR-30 based shRNA entry vector pEN_TRmiRc2 with dsRed2 co-expression. pEN_TRmiRc2 was a gift from Iain Fraser (Addgene plasmid #25750). The following sequences within the Rab27A 3′UTR were targeted: #1: CCAGCTCAATGTCTTTGAGTAT and #2: CGCTCAATGTCTTTGAGTATTA. The resulting fragment was cloned into the pLenti CMV Puro DEST expression vector, a gift from Eric Campeau (Addgene plasmid #17452), using the Gateway LR Clonase II Enzyme (Invitrogen/Life Technologies, Carlsbad, CA). The lentiviral expression vectors were used to generate VSVg pseudotyped lentivirus. Virus production and infection of cells was performed similarly as before [44]. The 3rd generation packaging plasmids pMD2.G (Addgene plasmid #12259); pMDL/RRE g/p (Addgene plasmid #12251), and pRSV-Rev (Addgene plasmid #12253) were a gift from Didier Trono. The packaging plasmids were co-transfected with the lentiviral expression vector into human embryonic kidney 293 T cells using the polyethylenimine (Polysciences, Inc. Warrington, PA) transfection method to produce replication deficient lentivirus. After 48 and 72 h of transfection, supernatants were pooled, filtered through a 0.45-μm MCE membrane and concentrated using polyethylene glycol (Sigma-Aldrich) [45, 46]. MCF7 and MDA-MB-231 were infected with lentivirus in the presence of 8 μg/ml polybrene (Sigma-Aldrich). Approximately 48 h post-infection cells were selected for gene transfer by treating with 1 μg/ml puromycin (InvivoGen, San Diego, CA).

### Fluorescent spectrometry, microscopy and cell imaging

CD63-GFP (copGFP) levels were measured on BioTek Synergy HT plate reader (BioTek Instruments, Inc. Winooski, VT) and detected by excitation at 485 nm and emission at 528 nm. Endothelial cells were analyzed by fluorescence microscopy using the Operetta High Content Imaging System (PerkinElmer, Waltham, MA). Endothelial cells were plated on Matrigel in Cell Carrier-96 plate from (PerkinElmer) at a density of 10,000 cells per well. Whole exosomes were labeled by combining 1 mL of whole exosomes isolated by ultracentrifugation with 20 μM acridine orange (AO, Molecular Probes/Life Technologies). These exosomes were incubated in the dark at room temperature for 1 h, diluted in 20 mL of PBS, and ultracentrifuged at 100,000 × *g* for 1 h. The exosome pellet was then resuspended in PBS. AO was detected by excitation at 460 nm, emission at 650 nm for red fluorescence.

### Endothelial tube formation assay (*in vitro* angiogenesis)

Endothelial tube formation was measured utilizing the *in vitro* Angiogenesis Assay Kit (Millipore, Billerica, MA) as we have previously described [32]. EA.hy926 (1x10^4^) cells were resuspended in EBM-2 (Lonza Group, Walkersville, MD) medium and plated onto the polymerized extracellular matrix. The formation of endothelial tubes was observed microscopically and photographed after 17–24 h incubation. Tube formation was quantified by branch point counting as specified by the manufacturer.

### VEGF secretion

Secretion of VEGF from MCF-7 cells was determined using an ELISA kit as previously reported [32]. Cells were seeded into 6-well plates at a density of 1x10^6^ cells/well and treated with clofibrate or troglitazone for 4 h prior to placement into the hypoxia chamber for 16 h. The culture medium was then collected, and the level of VEGF in the medium was analyzed following the manufacturer’s instructions.

### RNA extraction

Total RNA was extracted using the TRIzol reagent (Invitrogen/Life Technologies) following the manufacturer’s protocol. RNA concentration was quantitated using the NanoDrop ND-100 Spectrophotometer (NanoDrop Technologies, Wilmington, DE) and the quality was assessed using the Agilent 2100 Bioanalyzer (Agilent Technologies, Palo Alto, CA), according to the manufacturer’s protocol.

### Small RNA library preparation and next generation sequencing

A small RNA library was prepared from 100 ng of total RNA using the TruSeq Small RNA Preparation Kit (Illumina, San Diego, CA). The MiSeq next generation sequencer (Illumina) was used to sequence the resulting cDNA (2x25bp, 50 cycles). The reads were mapped onto the human genome (hg18 build [47]) and intersected with microRNAs (mirBase.org [48]) using GeneSifter Software (PerkinElmer, Santa Clara CA). Reads were normalized to the mapped reads and significant differences in reads were determined using the Likelihood Ratio Test and the Benjamini and Hochberg post-test. A *p* < 0.05 is considered significant. P-values close to 0 are represented as 1.0E-20. The RNA sequencing data discussed in this publication have been deposited in NCBI’s Gene Expression Omnibus [49] and are accessible through GEO Series accession number GSE70432 (http://www.ncbi.nlm.nih.gov/geo/query/acc.cgi?acc=GSE70432).

### Quantitative real-time reverse transcription PCR

For microRNA expression analysis complementary DNA from 5 ng of total RNA was synthesized by the addition of a microRNA specific 5X reverse transcription stem-loop primer and the TaqMan microRNA Reverse Transcription Kit, according to the manufacturer’s instructions. Real-Time PCR was performed by diluting the cDNA product in 2X TaqMan Universal Master Mix II (with UNG) and 20X TaqMan microRNA Expression Assay for each mature microRNA to be measured: miR-21 (ID:000397), miR-23b (ID:00400), miR-27b (ID:000409), miR-320b (ID:002844), and let-7a (ID:000377). All reagents and primers were from Life Technologies. The small ribonuclear RNA RNU6B (ID:001093) served as a microRNA expression normalization control for cellular microRNA expression analysis. However, we found RNU6B to be unreliable for exosome microRNA expression normalization, therefore we used a synthetic *Caenorhabditis elegans* miR-54 (cel-miR-54) RNA oligonucleotide (Integrated DNA Technologies, Coralville, IA) as a spike-in control. Cel-miR-54 has previously been shown not to affect human microRNA detection [50]. The cel-miR-54 (0.25 nM) oligonucleotide was spiked into each RNA sample prior to complementary DNA synthesis and Real-Time PCR was performed using the TaqMan microRNA assay (ID:001361, Life Technologies). microRNA target gene expression was measured by generating cDNA from 200 ng of total RNA using the iScript cDNA Synthesis kit (Bio-Rad, Hercules, CA). The synthesized cDNA was diluted in 2X iTaq Universal SYBR Green Supermix (Bio-Rad, Hercules, CA) and combined with 4uM of each forward and reverse primer. Specific primer sequences used are as follows NRP1, forward 5′-CCTTCTGCCACTGGGAACAT-3′ and reverse 5′-TTGCCATCTCCTGTGTGATCC-3′; AMOTL1, forward 5′-CGAGGGACTGAACTAGCCAT-3′ and reverse 5′-AGGGGACCCTTTCACCG-3′; PRDX1, forward 5′-CTGGTTGAACCCCAAGCTGATA-3′ and reverse 5′-CAGCTGTGGCTTTGAAGTTGG-3′; PLAU, forward 5′-CGACTCCAAAGGCAGCAATGA-3′ and reverse 5′-TGGACACACATGTTCCTCCATT′3′; ETS2, forward 5′-CTCACCAACAATTCTGGGACTC-3′ and reverse 5′-CACATCCAGCAAGGACGACT-3′; and the normalization control 36B4, forward 5′-ATCAACGGGTACAAACGAGTCCTG-3′ and reverse 5′- AAGGCAGATGGATCAGCCAAGAAG-3′. PCR reactions were run on the Bio-Rad CFX 96 Real-Time PCR (Bio-Rad, Hercules, CA) instrument under the following conditions: hold at 95 °C for 10 min, then 40 cycles of 95 °C for 15 s and 60 °C for 1 min. Relative gene expression was assessed using the differences in normalized Ct (ΔΔCt method) after normalization to RNU6B (cellular microRNA) or cel-miR-54 (exosome microRNA). Fold changes were calculated using 2^-ΔΔCt^.

### microRNA mimic transfection

miRIDIAN microRNA mimics (50 nM) were transfected into endothelial cells using the DharmaFect Duo reagent (Dharmacon/GE Healthcare, Lafayette, CO) according to manufacturer’s protocol.

### Statistical analysis

All statistical analyses were completed using GraphPad Prism software (GraphPad Software, Inc. La Jolla, CA). When appropriate a Student’s t-test or One-way analysis of variance (ANOVA) with Dunnett’s post-test was used to determine statistically significant differences among control and experimental groups, with a *p* < 0.05 or lower as the level of significance.

## Additional files

Additional file 1:
**Next generation small RNA sequencing data.** This Excel file contains the microRNA reads and pairwise analysis results for the control and DHA treated MCF7 cells and exosomes. Additional file 2:
**CD63-GFP expression in breast cancer cells.** This file contains microscopic images confirming CD63-GFP expression in breast cancer cells. Additional file 3:
**Transfer of exosome microRNA from MCF7 exosomes to endothelial cells.** This file contains microRNA expression data confirming exosome microRNA transfer from MCF7 exosomes to endothelial cells.
