# Supplementary material for: Exosome-mediated microRNA signaling from breast cancer cells is altered by the anti-angiogenesis agent docosahexaenoic acid (DHA)
Source: Mol Cancer. 2015 Jul 16;14:133. doi: 10.1186/s12943-015-0400-7 (PMC4504101; doi:10.1186/s12943-015-0400-7)
Supplement: Additional file 2: — CD63-GFP expression in breast cancer cells. This file contains microscopic images confirming CD63-GFP expression in breast cancer cells. [file 12943_2015_400_MOESM2_ESM.pptx]

## Slide 1
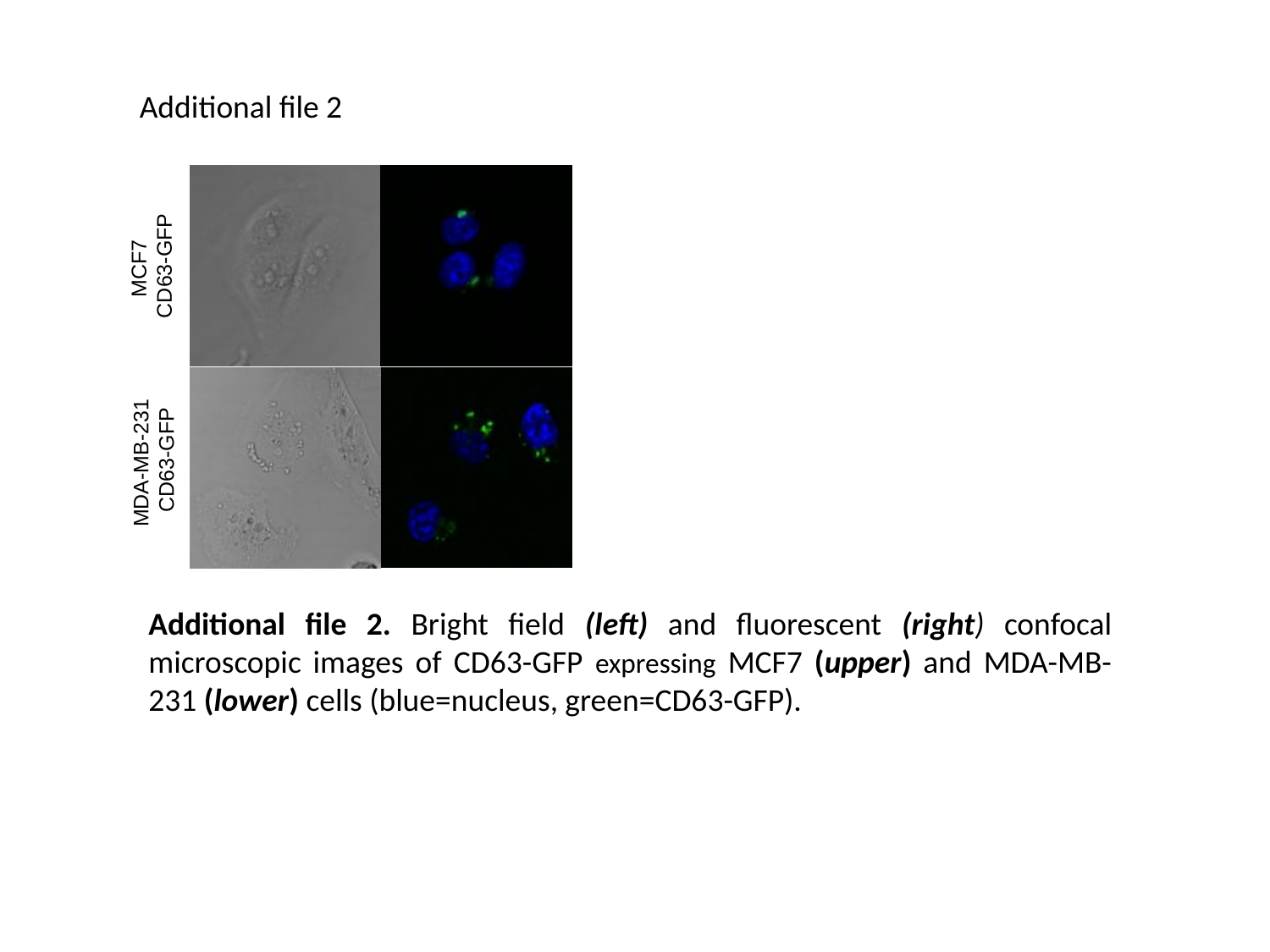

Additional file 2
MCF7
CD63-GFP
MDA-MB-231
CD63-GFP
Additional file 2. Bright field (left) and fluorescent (right) confocal microscopic images of CD63-GFP expressing MCF7 (upper) and MDA-MB-231 (lower) cells (blue=nucleus, green=CD63-GFP).
