# Supplementary material for: Exosome-mediated microRNA signaling from breast cancer cells is altered by the anti-angiogenesis agent docosahexaenoic acid (DHA)
Source: Mol Cancer. 2015 Jul 16;14:133. doi: 10.1186/s12943-015-0400-7 (PMC4504101; doi:10.1186/s12943-015-0400-7)
Supplement: Additional file 3: — Transfer of exosome microRNA from MCF7 exosomes to endothelial cells. This file contains microRNA expression data confirming exosome microRNA transfer from MCF7 exosomes to endothelial cells. [file 12943_2015_400_MOESM3_ESM.pptx]

## Slide 1
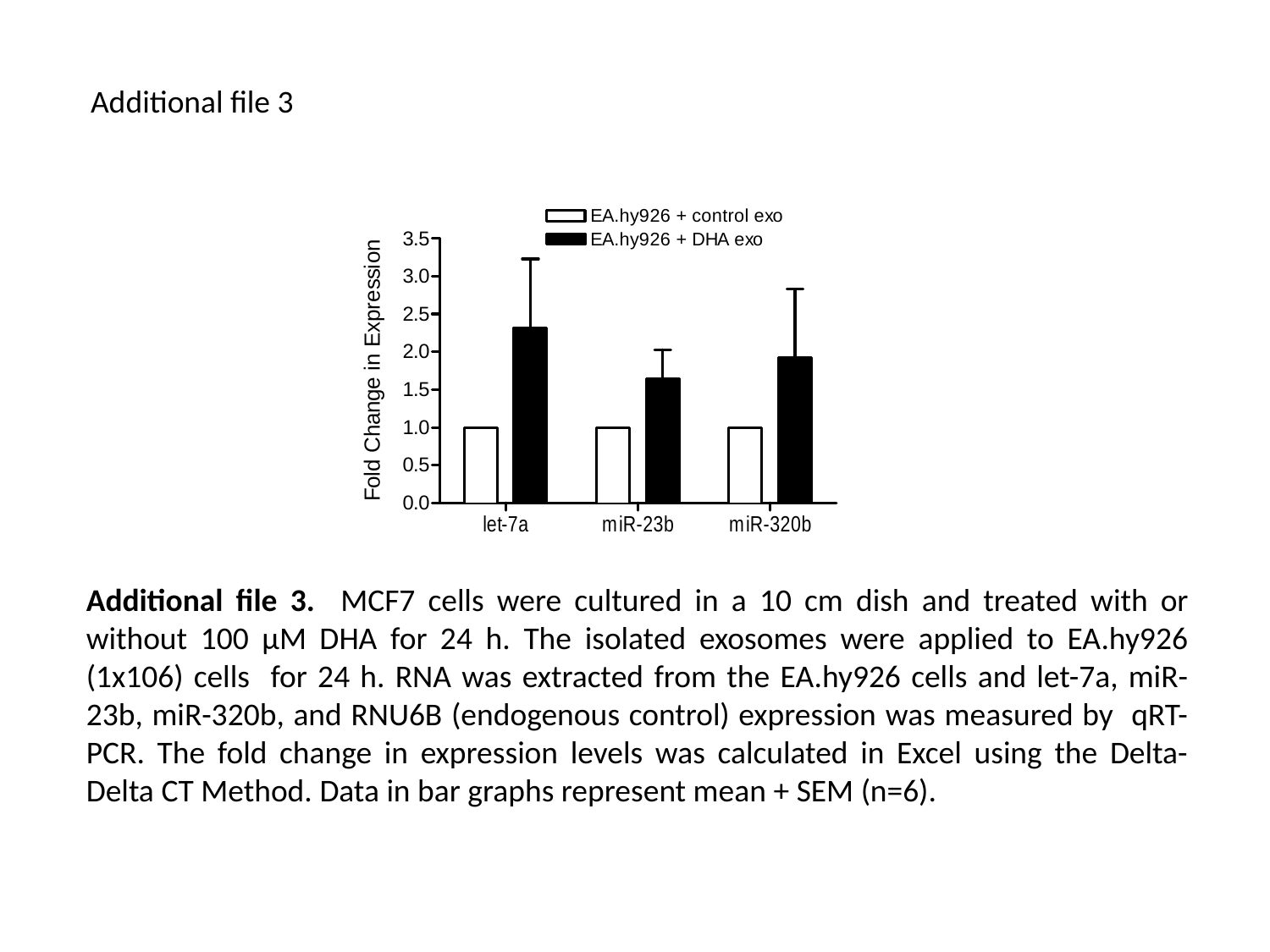

Additional file 3
Additional file 3. MCF7 cells were cultured in a 10 cm dish and treated with or without 100 µM DHA for 24 h. The isolated exosomes were applied to EA.hy926 (1x10^6^) cells for 24 h. RNA was extracted from the EA.hy926 cells and let-7a, miR-23b, miR-320b, and RNU6B (endogenous control) expression was measured by qRT-PCR. The fold change in expression levels was calculated in Excel using the Delta-Delta CT Method. Data in bar graphs represent mean + SEM (n=6).
